# Surface Display Technology for Biosensor Applications: A Review

**DOI:** 10.3390/s20102775

**Published:** 2020-05-13

**Authors:** Min Park

**Affiliations:** 1 Major in Materials Science and Engineering, Hallym University, Chuncheon, Gangwon-do 24252, Korea; minpark@hallym.ac.kr; Tel.: +82-33-248-2363; 2 Integrative Materials Research Institute, Hallym University, Chuncheon, Gangwon-do 24252, Korea

**Keywords:** biosensors, surface display, molecular recognition layer, molecular display, phage display, bacterial surface display, yeast cell surface display

## Abstract

Surface display is a recombinant technology that expresses target proteins on cell membranes and can be applied to almost all types of biological entities from viruses to mammalian cells. This technique has been used for various biotechnical and biomedical applications such as drug screening, biocatalysts, library screening, quantitative assays, and biosensors. In this review, the use of surface display technology in biosensor applications is discussed. In detail, phage display, bacterial surface display of Gram-negative and Gram-positive bacteria, and eukaryotic yeast cell surface display systems are presented. The review describes the advantages of surface display systems for biosensor applications and summarizes the applications of surface displays to biosensors.

## 1. Introduction

**Figure 1:**
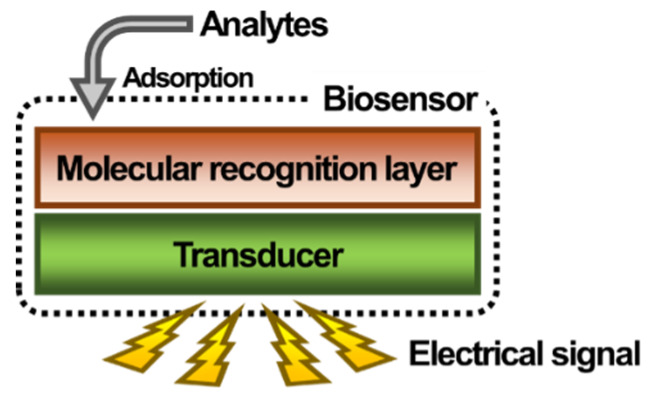
Schematic of a biosensor.

A biosensor is an analytical device that selectively provides a quantitative response to a single or several analytes among thousands of compounds in complex samples such as physiological fluids including blood, cerebrospinal fluid, urine, and saliva [1,2]. As shown in Figure 1, biosensors are generally composed of three main parts: a molecular recognition layer, a transducer, and a signal generator [3]. In general, the molecular recognition layer is produced by the immobilization of a bioreceptor on the surface of the transducer to allow specific binding of a target analyte [1,4]. After the bioreceptor binds the analyte, a measurable signal can be generated by the transducer through changes in the thickness, weight, refractive index, or the structure of the molecule via chemical reaction [5,6,7,8,9,10,11,12,13]. The molecular recognition layer is especially important for achieving specific detection in biomedical analyses. To achieve specific and strong binding of the analyte to the biosensor, various bioreceptors such as antibodies, aptamers, enzymes, and peptides have been used [14,15,16,17,18,19]. The physiological fluids mentioned above are complex mixtures of thousands or millions of ions, proteins, nucleic acids, and cells; therefore, the specific binding of a target analyte in physiological fluid is one of the most important challenges for increasing the sensitivity and reducing the noise of biosensors [20].

Cells are the basic units of all organisms, including humans, and are enclosed within a membrane. The cell membrane comprises various membrane proteins and other biomolecules that interact via covalent or non-covalent bonds [21,22]. These membrane proteins play important roles in cell signaling, influencing the enzymatic environment, molecular transport, and cell identification [23,24,25]. Among the functions of membrane proteins, receptor proteins can recognize signaling molecules, and this recognition is very similar to the role of the molecular recognition layer in biosensors [26]. By using these membrane proteins and protein engineering, the designed protein can be displayed on the cell surface. Unlike the general protein expression mechanism, membrane protein-linked proteins are anchored and displayed on the cell surface, and this surface display technology can be utilized for translocation, biocatalysis, drug screening, library screening, etc., [27,28,29,30]. Affinity proteins are the main targets of surface displays, and these proteins can potentially be applied as molecular recognition layers. In addition, these surface display technologies has advantage that the surface displaying particles or cells can be act as a biological matrix to detect the target analyte from. In this review, various types of surface display technologies are introduced, and their application in biosensors is discussed. In detail, phage displays, bacterial surface displays, and eukaryotic cell surface displays are introduced, and the applications of these systems to biosensors are summarized. This review provides a brief and concise discussion of the application of surface display technology in the molecular recognition layer of biosensors.

## 2. Phage Display-Based Biosensors

Surface expression systems were initially based on phage display systems. Phages are viruses that infect bacterial cells. Phage display was first introduced by Smith, when he inserted a foreign DNA fragment of a peptide library into the minor coat protein of the filamentous bacteriophage, pIII [31]. For the phage display of filamentous bacteriophage, the major coat protein pVIII and minor coat proteins pIII and pVI are the major recombinant targets [32]. Various libraries have been displayed on phages, and library-displaying phages can be selectively screened using biopanning [33,34,35]. Phage display has been widely used for affinity screening from random libraries because phage libraries have high diversity. The affinity peptides from library screening can be utilized as bioreceptors for the molecular recognition layer [36,37].

Various studies have investigated the application of phage display systems to biosensors. In most studies, random peptides or scFv antibodies libraries were displayed on the phage particles and screened to find the affinity peptide or protein by biopanning. After biopanning, the selected peptides or phage particles were used to form a molecular recognition layer for biosensor applications. The biosensor applications of phage display systems and their performance are summarized in Table 1.

**Figure 2:**
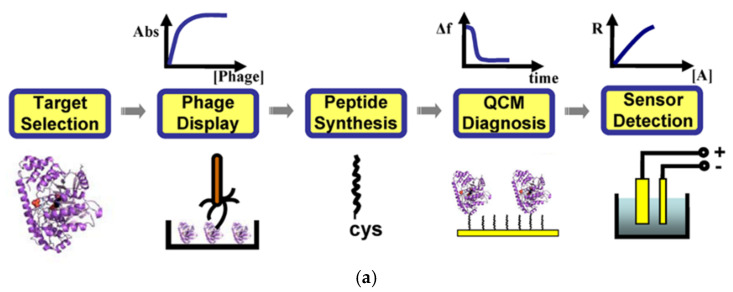

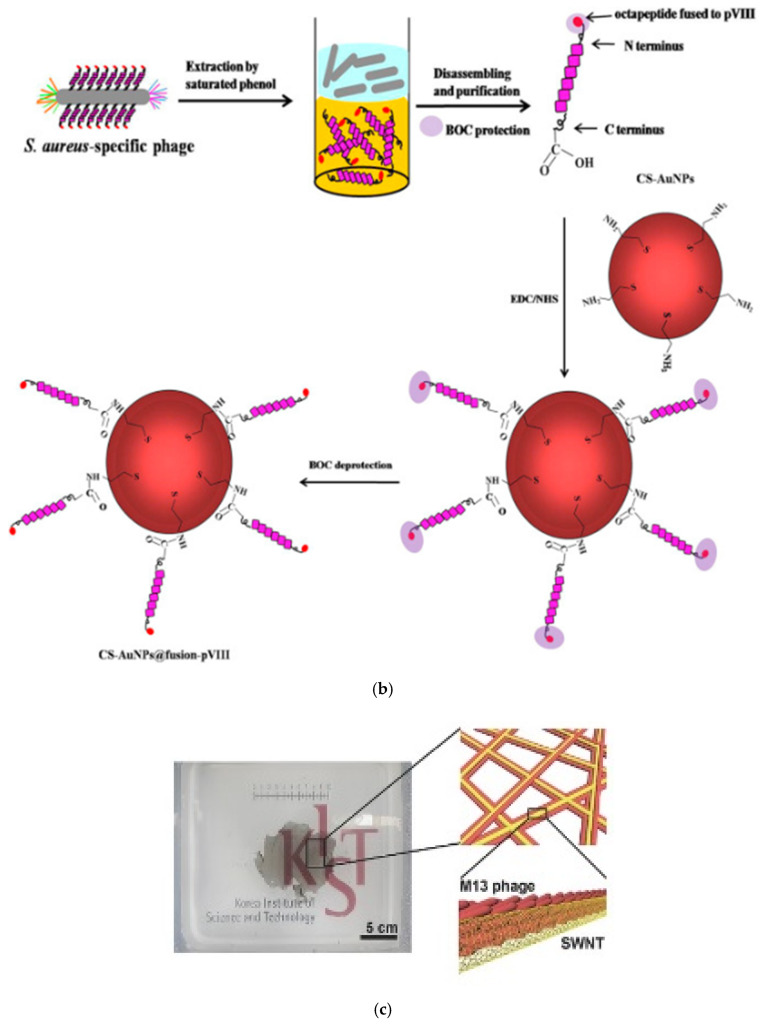
Examples of phage display-based biosensors. (**a**) Bioreceptor screening and application to a biosensor using peptide library-displaying bacteriophage. Reproduced with permission from [38]. Copyright (2010). Plos. (**b**) Fabrication of pVIII protein from phage-immobilized gold nanoparticles (GNP). Reproduced with permission from [40]. Copyright (2016) Elsevier. (**c**) Carbon nanotube (CNT) nanomesh using the M13 phage as a biological glue. Reproduced with permission from [42]. Copyright (2016) Wiley and Sons, Inc.

Wu et al. developed a peptide-based molecular recognition layer using electrochemical impedance spectroscopy (EIS) and a quartz crystal microbalance (QCM) biosensor to detect aminotransferase (ALT) and troponin I (TnI) (Figure 2a) [38,39,45]. Peptides with the target affinity were selected by biopanning from the random peptide library displaying the M13 phage and immobilized on the biosensor surface as a molecular recognition layer. By using the affinity peptides, a QCM and EIS biosensors were used to detect ALT and TnI. In these studies, the displayed phage was only used to screen the affinity peptide for specific analyte binding, and bacteriophage particles were not directly applied to the biosensors.

Liu et al. developed a colorimetric biosensor by utilizing the pVIII protein isolated from a phage (Figure 2b [40]. The *Staphylococcus aureus* binding phage with the f8/8 landscape phage library was selected, and the pVIII protein of the selected phage was isolated and purified. The isolated specific pVIII protein was immobilized on amine-functionalized gold nanoparticles (GNPs) via EDC/NHS chemistry, and the protein-immobilized gold nanoparticles were utilized for the detection of *S. aureus* by colorimetry. Rapid and one-step biosensing was realized via the developed method. In these studies, the major coating proteins of the phages were exploited for biosensing.

In another approach, the entire M13 phage particle was utilized for the fabrication of a carbon nanotube (CNT)-based nanomesh [41] and applied to enzyme-based electrochemical biosensors (Figure 2c) [42]. In this method, single-walled carbon nanotube (SWNT)-binding phages were selected from phages displaying the pVIII peptide library by biopanning. After affinity confirmation, a conductive and flexible nanomesh was assembled by using the phage particles and SWNTs [41]. The fabricated nanomesh was integrated into a flexible microarray and implanted in the skull of a mouse. In comparison with the bare electrode, the microarray allowed the detection of high-density electroencephalography (EEG) signals and significantly increased high-frequency brain signal (HFBS) levels. This phage display-based CNT nanomesh was applied to the fabrication of an enzyme-based biosensor employing direct electron transfer (DET) [42]. This DET biosensor based on phage display was confirmed to be suitable for the detection of glucose, cholesterol, lactate, peroxide, galactose, and catechol/catechol amine by changing the immobilized enzymes.

Nanduri et al. [43] utilized phage particles as molecular recognition elements. Phages with β-galactosidase affinity were selected and whole viral particles were immobilized on the surface of the QCM biosensor by simple physical adsorption. The dissociation constant (*K_d_*) for the molecular recognition layer based on phage display was similar to that of the monoclonal antibody-based layer. From this study, phage particles were confirmed to be suitable components of the molecular recognition layer.

Unlike the studies described above, Nanduri et al. displayed antibodies and utilized phage particles to detect *Listeria monocytogenes* (*L. monocytogenes*) using an SPR biosensor [44]. A single-chain variable fragment (scFv) displaying phage particles with specific affinity for *L. monocytogenes* was selected from a random scFv-displaying phage library [46] and immobilized on the surface of the SPR biosensor to form a molecular recognition layer.

## 3. Bacterial Surface Display for Biosensor Applications

Phage display technology has been widely used for the expression of libraries, antibodies, and specific ligands. However, the fusion of larger-sized targets in phage display systems is restricted by size limitations [47,48]. Cell surface displays based on bacterial cells offer the advantages of high yield, high productivity, and capability to display large-sized targets [48,49,50,51]. Bacteria are prokaryotes and biological cells, and can proliferate by binary fission. Because of these advantages, bacterial cells are the systems most frequently used for the expression of recombinant proteins [52]. For this reason, bacterial surface display is also actively researched and applied to biosensors [53]. In this section, bacterial surface displays for biosensor applications are discussed, and examples are presented.

### 3.1. Gram-Negative Bacteria

Gram-negative bacteria are not stained by crystal violet; the cell envelopes of Gram-negative bacteria are composed of an inner membrane, peptidoglycan, and outer membrane [54]. Among Gram-negative bacteria, *Escherichia coli* (*E. coli*) has been widely used because of its genetic availability and high transformation efficiency [29,55]. *E. coli* cells have been employed in various surface display strategies. Outer membrane proteins, lipoproteins, fimbria proteins, and flagellar proteins have been used for the surface display of target proteins on cells [56,57,58,59]. For the successful surface display of Gram-negative bacteria, the expressed proteins should cross the inner membrane and become anchored on the outer membrane [60]. For these reasons, various secretion mechanisms have been used for Gram-negative surface display [51,61].

Bacterial surface display systems offer the possibility of displaying larger molecules, including active enzymes, as their main advantage. In this respect, various studies of Gram-negative bacterial surface displays are related to the display of active enzymes. Such enzyme-displaying bacteria are valuable not only as molecular recognition molecules, but also as signal-generating molecules. Electrochemical biosensors can be fabricated based on these enzyme-surface-displaying bacteria. In addition, affinity molecules such as Z-domain or streptavidin can also be displayed on the surface of Gram-negative bacterial cells. By controlling the orientation of the displayed affinity molecules, improved performance of the biosensor was obtained. The biosensor applications of Gram-negative bacterial surface display systems are summarized in Table 2.

**Figure 3:**
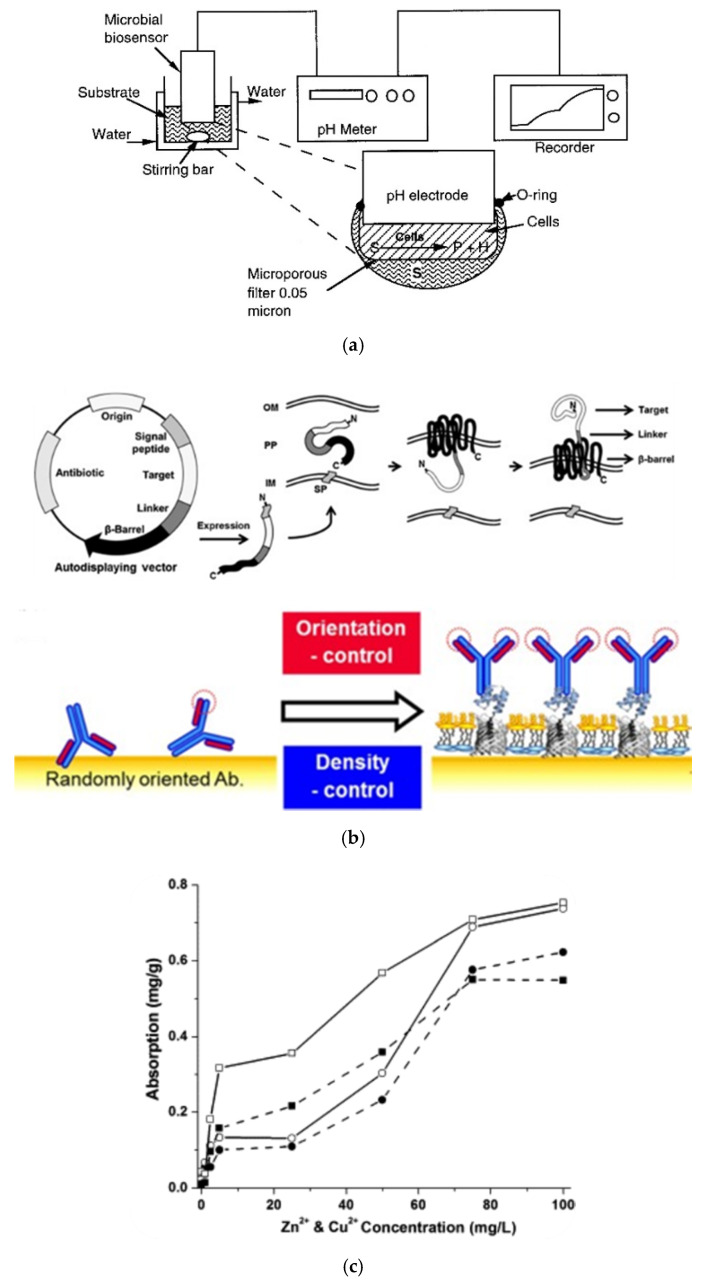

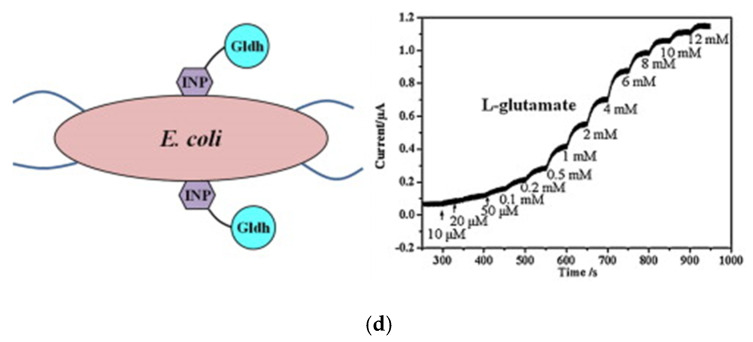
Biosensors based on Gram-negative bacterial surface display. (**a**) pH meter based on surface-displayed immobilized *E. coli*. Reproduced with permission from [62]. Copyright (1998) American Chemical Society. (**b**) Autodisplay of Z-domains and orientation control of the immunoaffinity layer based on autodisplayed Z-domains. Reproduced with permission from [9]. Copyright (2018) Elsevier. (**c**) Bioadsorption of heavy metal ions by surface-displayed *E. coli* cells: *E. coli* cells with (line) and without (dashed-line) surface-displaying vector were induced by Zn^2+^ (circle) and Cu^2+^ (square). Reproduced with permission from [68]. Copyright (2012) Elsevier. (**d**) Amperometric glutamate biosensor based on surface-displayed *E. coli* cells. Reproduced with permission from [65]. Copyright (2015) Elsevier.

Richins et al. displayed active organophosphorus hydrolase (OPH) on the surface of *E. coli* through the Lpp(Braun’s lipoprotein)-OmpA (outer membrane protein A) fusion system for parathion and paraoxon degradation [75] and Mulchandani et al. fabricated a biosensor based on immobilized OPH displaying *E. coli* on a pH electrode (Figure 3a) [62]. After optimization, the fabricated biosensor was used to measure the concentration of organophosphates (OPs), i.e., paraoxon, methyl parathion, and diazinon. The limit of detection (LOD) for all analytes was lower than 5 μM, which is comparable to those of enzyme-based biosensors. Tang et al. [76] achieved the surface display of OPH using ice nucleation protein (INP) and applied this system to an electrochemical biosensor [63]. OPH surface-displaying *E. coli* cells were immobilized on the electrode with mesopore carbons and the concentration of the OPs (paraoxon, parathion, and methyl parathion) was measured by amperometry using the surface-displaying *E. coli* cell-based biosensor. From the measurement, the linear detection range and LOD were calculated to be 0.05–25 μM and 9.0 nM for paraoxon; 0.05–25 μM and 10.0 nM for parathion; and 0.08–30 μM and 15.0 nM for methyl parathion, respectively.

Liang et al. fabricated an electrochemical biosensor by using a bacterial surface display (Figure 3d) [64,65]. First, glucose dehydrogenase (GDH) was surface displayed on the surface of *E. coli* cells by using INP [64]. Surface-displayed *E. coli* cells were immobilized on the surface of a glassy carbon electrode (GCE). An amperometric glucose biosensor was fabricated by employing the *E. coli* cell-immobilized electrode. The LOD for glucose was calculated to be 4 μM, and this glucose biosensor was confirmed to be applicable to real samples. Glutamate dehydrogenase (GluDH) was surface displayed on the outer membrane of *E. coli* cells using INP, and an amperometric glutamate biosensor was fabricated using surface-displayed *E. coli* cells [65]. From the glutamate measurement, the LOD of the fabricated biosensor was calculated to be 2 μM.

Zhang et al. fabricated a voltammetric biosensor based on the surface display technology [66]. In this study, laccase was surface displayed through the INP variant (*inaQ-N*), and surface-displayed cells were immobilized on the surface of the electrode to fabricate the biosensor. The optimal conditions for catechol detection, including the cell loading, pH, and scan rate were 4 × 10^8^ cells, 3.0, and 100 mV S^–1^, respectively. The linear range and LOD for catechol detection were calculated to be 0.5, 300.0 and 0.1 μM, respectively. In addition, the fabricated biosensor based on the surface display technology was confirmed to be suitable for the detection of catechol in real samples, red wine, and tea.

A dual surface display employing a gold-binding peptide (GBP) and core streptavidin (cSA) on the surface of *E. coli* was investigated by Park et al. [67]. In that study, GBP was surface displayed via FadL and utilized for the immobilization of surface-displayed *E. coli* cells on the gold surface of the SPR biosensor. cSA was surface displayed via OprF and used as the analyte recognition molecule. By utilizing the dual surface display system, whole *E. coli* cells were immobilized on the surface of the SPR biosensor to form a molecular recognition layer, and biotinylated HRP was detected.

Ravikumar et al. developed a bacterial cell-based fluorescence biosensor for heavy metal (zinc and copper) detection [68]. Green fluorescent protein (GFP) and red fluorescent protein (RFP) were expressed by induction with Zn^2+^ and Cu^2+^, respectively, and the fluorescence biosensor was fabricated based on the whole *E. coli* cell. This biosensor was extended by using the surface display of a zinc binding peptide (ZBP) and copper binding protein (CBP) by outer membrane protein C (OmpC) (Figure 3c).

Autodisplay technology, a type of surface display for Gram-negative bacteria, has been developed based on the secretion mechanism of the autotransporter family proteins, where the expressed protein does not require any artificial treatment to pass through the inner membrane [1,77,78]. This technology also has been used to display affinity proteins, enzymes, libraries, and other species, and has been utilized in various applications including biosensors, biocatalysts, drug screening [27,79,80]. Jose et al. expressed immunoglobulin(Ig) G binding Z-domains [81] on the outer membrane of *E. coli* using autodisplay technology by utilizing the adhesion involved in diffuse adherence (AIDA-I), and applied this system to signal amplification in a SPR biosensor (Figure 3b) [69]. By signal amplification, the LOD for cardiac myoglobin detection was improved 10-fold compared to that without amplification. The Z-domains autodisplaying *E. coli* cells were immobilized on the SPR biosensor to construct a molecular recognition layer [70]. From the detection of C-reactive protein (CRP), an acute phase inflammatory biomarker, the cut-off value was calculated to be improved by 5-fold. Because Z-domains can bind the F_c_ region of IgG, orientation control can be realized and the performance of the immunosensor can be improved [1]. The outer membrane of Z-domains autodisplaying *E. coli* was isolated by lysozyme and Triton X-100 treatments [82] and layered on a 2-dimensional substrate [83]. This outer membrane layer with autodisplayed Z-domains was used as the immunoaffinity layer of a SPR biosensor after antibody treatment [71,72]. The performance of the SPR biosensor for detection of CRP and human IgG (hIgG) was improved by 100-fold and 10-fold through orientation control based on autodisplay technology, respectively. In addition, this immunoaffinity layer based on autodisplay technology could be reused after regeneration by acid treatment [84].

### 3.2. Gram-Positive Bacteria

The cell envelope of Gram-positive bacteria is composed of a plasma membrane and a thick outer peptidoglycan layer that is stained by crystal violet [54,85]. Because of the thick and rigid peptidoglycan layer, Gram-positive bacteria are considered to be suitable for cell-based applications such as whole-cell catalysts and whole-cell adsorbents [29]. For this reason, the surface display of Gram-positive bacteria has also been actively studied and applied in various fields, including biosensors [86,87]. Unlike Gram-negative bacterial surface display, most Gram-positive surface-displaying proteins are immobilized on the cell wall by covalent bonding [51,86].

Kronqvist et al. developed a screening system utilizing both phage display and bacterial surface display of Gram-positive cells [73]. To select the affinity protein (affibody) with tumor necrosis factor-alpha (TNF-α), pre-enrichment was performed in one round of screening using a phage-displayed library. After screening, the staphylococcal display vector containing albumin-binding protein (ABP) from streptococcal protein G was constructed using the sequences from pre-enrichment. After vector transformation into *Staphylococcus carnosus*, surface-displaying cells with TNF-α affinity were selected by sorting using flow cytometry. Selected surface-displaying cells were immobilized on the SPR biosensor to determine the *K_d_* values. A similar approach was used to screen the affibody with ErbB3, an epidermal growth factor of transmembrane tyrosine kinase receptor [74]. From the SPR biosensor analysis, the *K_d_* and *K_a_* (association constant) values were calculated to be 0.7 nM and 1.9 × 10^6^ M^–1^∙s^–1^, respectively. In those studies, staphylococcal surface-displaying affibodies were applied to the SPR biosensor, not for the fabrication of surface display-based biosensors, but for calculation of the affinity constant of screened affibodies. The molecular recognition layer was constructed based on the immobilized staphylococcal surface-displaying cells, and biosensing of target analytes could be accomplished.

## 4. Eukaryotic Yeast Cell Surface Display for Biosensor Applications

Yeasts are eukaryotic, unicellular microorganisms that are encapsulated within rigid cell walls, where the cell walls are mainly composed of mannoproteins and β-glucan [88]. For the surface display of a target protein on the surface of yeast cells, glycosylphosphatidylinositol (GPI)-anchored proteins such as glucanase-extractable mannoproteins, including agglutinin and flocculin, have been widely utilized [22,89]. In comparison with phage display and bacterial surface display, yeast cell surface display is advantageous in terms of safety and genetic engineering [90,91]. Yeast cells are widely used in the food and pharmaceutical industries because of their safety. In addition, yeast cells are eukaryotic cells that allow folding, disulfide isomerization, and glycosylation of expressed homologous proteins, especially complex eukaryotic proteins [92]. These surface-engineered yeasts are also called “arming yeasts” [93,94].

Yeast surface display systems offer the possibility of displaying complex eukaryotic proteins on the surface of safe microorganisms, i.e., yeast. Various types of proteins, such as fluorescent proteins, affinity proteins, and enzymes, have been displayed on yeast cell surfaces. Yeast cell surfaces displaying fluorescent proteins have been applied in optical biosensors, and enzyme surface displaying yeast cells were applied to electrochemical and optical biosensors. The biosensor applications of yeast cell surface displays are summarized in Table 3.

Tanaka et al. developed a glucose monitoring system based on the yeast cell surface display of a fluorescent protein [95]. GFP was fused with the C-terminal half of α-agglutinin for surface display on *Saccharomyces cerevisiae* (*S. cerevisiae*). Using the glucose-inducible promoter, GAPDH, expression of the surface-displayed GFP was controlled for glucose monitoring by fluorescence measurement using whole yeast cells. This yeast cell surface display system was modified by adding another surface displaying vector [96]. The blue fluorescent protein (BFP)-displaying vector with the UPR-ICL promoter was activated in the absence of glucose. By co-transformation of the two surface-displayed vectors in *S. cerevisiae*, the green fluorescence arising in the presence of glucose was monitored, whereas the emission of the BFP was restored after the consumption of glucose. A similar system was utilized to monitor phosphate and ammonium ions [97]. Enhanced cyan blue fluorescent protein (ECFP) and enhanced yellow fluorescent protein (EYFP) sequences were inserted into surface-displaying vectors with PHO5 and MEP2 promoters, respectively. In those studies, proteins were surface-displayed on yeast cells when their fluorescence was induced by the monitoring target. Thus, yeast cell surface display technology was applied to fluorescence biosensors for monitoring the presence of a target.

**Figure 4:**
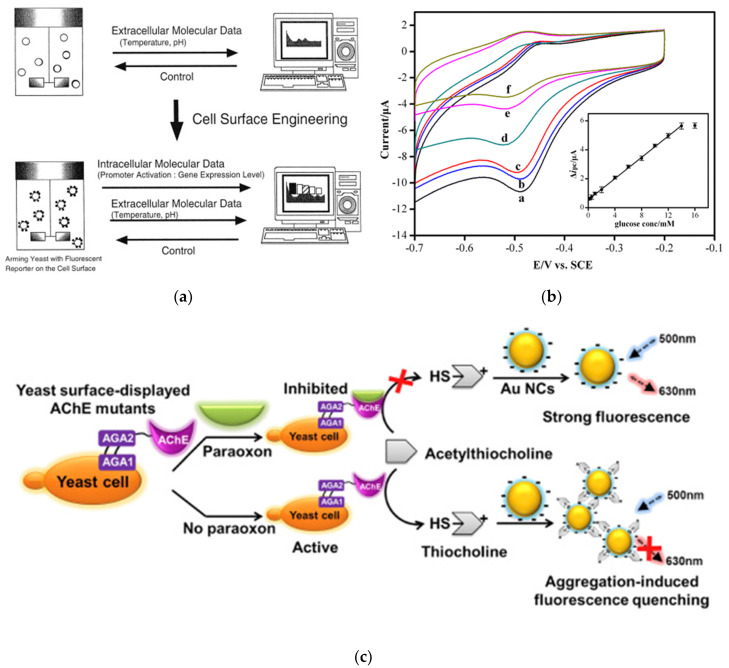
Biosensors based on yeast cell surface display. (**a**) Biomonitoring system based on surface display technology. Reproduced with permission [98]. Copyright (2003) Elsevier. (**b**) Electrochemical glucose biosensing based on glucose oxidase surface displaying yeasts. For the biosensing, glucose was spiked in PBS with different concentrations: 0.0 (a), 0.1 (b), 0.5 (c), 2.0 (d), 8.0 (e), and 12.0 (f) mM. Reproduced with permission [99]. Copyright (2013) American Chemical Society. (**c**) Paraoxon biosensing using AChE surface displaying yeasts. Reproduced with permission [101]. Copyright (2018) Elsevier.

A real-time monitoring system for bioprocessing using yeast cell surface display (Figure 4a) was also developed [98]. In that study, enhanced GFP (EGFP) was fused with agglutinin for the surface display, and the galactose-inducible promoter GAL1 was selected to regulate gene expression. The monitoring of foreign protein production (β-galactosidase for intracellular expression and human interferon-omega for extracellular expression) was established by simultaneous surface display of EGFP as a reporter with the same promoter.

Nakamura et al. displayed IgG-binding Z-domains on the surface of yeast cells by fusion with the C-terminal half of α-agglutinin [102]. ZZ-domain surface displaying yeast cells can be utilized as a solid support for enzyme-linked immunosorbent assay (ELISA) to detect human serum albumin and can be used for the affinity purification of IgG. That study demonstrated a ZZ-domain fused with EGFP and discussed the capability to study interactions, biosensing, and immunoassays [103]. These systems have great potential for application to biosensors, as described in Section 3.1.

Glucose oxidase (GOx) was surface-displayed on *S. cerevisiae* as a whole-cell biocatalyst, and this arming yeast was used as a glucose biosensor [99]. GOx was displayed on yeast cells as a fusion protein with α-agglutinin as an anchor motif. The surface-displayed GOx was confirmed to have good pH stability and thermostability with high specificity. GOx surface-displaying cells were immobilized on a GCE for electrochemical measurement. For glucose biosensing by cyclic voltammetry, the fabricated glucose biosensor showed a linear response range of 0.1–14 mM and LOD of 0.05 mM (Figure 4b). Furthermore, in real sample measurements, the developed biosensor showed good accuracy within 7% of the relative standard deviation.

Yeast surface display technology was applied to OP biosensing. Liang et al. expressed active acetylcholinesterase (AChE) through α-agglutinin as an anchor motif on the surface of yeast cells for spectrophotometric biosensing [100]. The surface-displayed AChE hydrolyzes acetylthiocholine chloride (ATCl) to form thiocholine, and this thiocholine reacts with 5,5′-dithiobis-2-nitrobenzoic acid (DTNB), which absorbs light at 415 nm. In the presence of OP pesticides such as paraoxon and parathion, the active site of AChE was inhibited, which prevented hydrolysis of ATCl, leading to no colorimetric response. Using the developed spectrophotometric biosensor, the LOD for paraoxon and parathion was calculated to be 0.49 and 12.8 nM with linear detection ranges of 1.8 nM–36.3 μM and 17–34.4 μM, respectively. The fabricated biosensor was also confirmed to be capable of measuring analytes in tap water, seawater, and sewage. Very recently, AChE surface-displaying yeast cells were utilized to fabricate a fluorescence biosensor [101]. As shown in Figure 4c, after hydrolysis of ATCl by the surface-displayed AChE, positively charged thiocholine with thiol groups was generated, leading to aggregation of gold nanoclusters (AuNCs) through electrostatic interactions, and the aggregated AuNCs quenched the fluorescence signal. For paraoxon detection, the LOD was calculated to be 3.3 × 10^−14^ M with a linear range of 1.0 × 10^−13^–1.0 × 10^−10^ M.

## 5. Conclusions and Perspectives

In this review, various types of surface display technologies such as phage display, bacterial surface display, and yeast cell surface display were discussed. Phage display is a useful tool for the display of libraries because of its high diversity. By using phage display and biopanning, affinity proteins can be screened with specific targets, and screened proteins or screened proteins displaying phage particles have been applied to the construction of molecular recognition layers. Examples of the application of phage display to biosensors are summarized in Table 1. In addition, other advantage of phage display is that phage can display several different targets. It means that phage can display both affinity protein in minor coat protein to immobilization of phage particle on the sensor surface and recognition protein in major coat protein to capture analytes. Phage display technology for the biosensor application will require more diversity and stability of displayed library to select new recognition molecules with high affinity.

The expression of larger proteins on the surface of phage particles is limited; thus, bacterial cells are utilized for expression of these proteins. By using bacterial cells, not only affinity proteins, but also active enzymes, can be displayed on the outer surface of the cells. Affinity proteins such as Z-domains can be applied to the construction of the immunoaffinity layer for biosensors. Surface-displayed active enzymes on bacterial cells may also be useful for the construction of a molecular recognition layer. In particular, the surface display of oxidoreductase is suitable for the fabrication of electrochemical biosensors. Bacterial surface displays and their biosensor applications are summarized in Table 2. Bacteria cells can proliferate by binary fission and single bacteria cell can be identified and sorted using the flow cytometry. It means that bacteria surface display system can offer easier process of screening than phage display. In addition, there is no size limitation of target protein in bacterial surface display. From these reason, bacterial surface display of peptides or antibodies libraries may play an important role in the biosensor applications. Additional advantage of bacterial surface display is that it offers membrane environment to surface displaying targets. Numerous enzymes only have activity in the membrane environment so utilization of these enzymes to the biosensor can be realized by bacterial surface display.

The surface display of a complex eukaryotic protein can be realized by yeast cells. Yeast cell surface display technology is also a good platform for the development of biosensing methods. Various proteins, including fluorescent proteins and enzymes, have been surface displayed on yeast cells and applied in biosensors. A summary of yeast cell surface display-based biosensors is presented in Table 3. In comparison to other surface display systems, yeast cell surface display has distinct merits of safety and ability to express complex eukaryotic proteins. As mentioned above, yeast cells can be used in the food and medicines so yeast cell surface display system can be applied to eatable biosensing systems, in vivo monitoring system, and so on. In addition, enzymatic biosensor utilizing eukaryotic proteins can be realized by yeast cell surface display.

Overall, this review sheds light on biosensing applications based on surface display technologies and their further potential for biosensor applications.

## Figures and Tables

**Table 1:** Examples of phage display-based biosensors and their performance.

Type of Library	Affinity Target	Detection Mode	Performance	Assay Time	Ref.
Peptide	Troponin I	QCM	Sensitivity: 18 Hz/(μg/mL) LOD: 0.11 μg/mL	-	[38]
		EIS	Sensitivity: 0.3 impedance/(μg/mL) LOD: 0.34 μg/mL	1 h	
	Aminotransferase	QCM	Sensitivity: 8.9 Hz/(μg/mL) LOD: 60 ng/mL	-	[39]
		EIS	Sensitivity: 142 impedance %/(μg/mL) LOD: 92 ng/mL	1 h	
	*S. aureus*	Colorimetry	LOD: 19 CFU/mL	30 min	[40]
	Mouse EEG signal	EIS	Contact impedance: 7.4 kΩ	-	[41]
	Glucose	Electrochemistry	Sensitivity: 107 μA/mM∙cm^2^ LOD: 10 μM	<10 s	[42]
	β-galactosidase	QCM	*K_d_* = 1.7 nM	100 s	[43]
scFv	*L. monocytogenes*	SPR	LOD: 2 × 10^6^ cfu/mL	-	[44]

**Table 2:** Examples of bacterial surface displays for biosensor applications.

Type of Target	Displayed Target	Display System	Detection Mode	Analyte	Performance	Assay Time	Ref
Enzyme	OPH	Lpp-OmpA	Potentiometry	Paraoxon	LOD: 2 μm	10 min	[62]
				Methyl parathion	LOD: 2 μM		
				Diazinon	LOD, diazinon: 5 μM		
		INP	Amperometry	Paraoxon	LOD: 9.0 nM	5 s	[63]
				Parathion	LOD: 10.0 nM		
				Methyl parathion	LOD: 15 nM		
	GDH		Amperometry	Glucose	LOD: 4 μM	2 s	[64]
	GluDH		Amperometry	Glutamate	LOD: 2 μM	-	[65]
	Laccase	*inaQ-N*	Voltammetry	Catechol	LOD: 0.1 μM Linear range: 0.5–300 μM	-	[66]
Affinity peptide or protein	GBP	FadL	SPR	Biotinylated HRP	0.62 % SPR angle change	20 min	[67]
	cSA	OprF					
	ZBP	OmpC	Fluorescence	Zn^2+^	0.74 mg/g adsorption	4 h	[68]
	CBP			Cu^2+^	0.75 mg/g adsorption		
	Z-domain	AIDA-I	SPR	myoglobin	10-fold improved LOD	1 h	[69]
				CRP	5-fold improved cut-off value	40 min	[70]
				CRP	100-fold improved LOD	35 min	[71]
				hIgG	10-fold improved LOD	40 min	[72]
	affibody	ABP	SPR	TNF-α	*K_d_*: 0.77 nM	20 min	[73]
			SPR	ErbB3	*K_d_*: 0.7 nM	11 min	[74]

**Table 3:** Examples of yeast cell surface displays for biosensor applications.

Displayed Target	Detection Mode	Analyte	Performance	Assay Time	Ref
GFP	Fluorescence	Glucose	Available for monitoring the presence of analyte	-	[95]
GFP, BFP		Glucose		-	[96]
ECFP, EYFP		Phosphate, ammonium ion		-	[97]
EGFP		Protein expression	Simultaneous displaying for protein expression monitoring	-	[98]
GOx	Voltammetry	Glucose	LOD: 0.05 mM Linear range: 0.1–14 mM	20 s	[99]
AChE	Spectrophotometry	Paraoxon	LOD: 0.49 nM Linear range: 1.8 nM–36.3 μM	15 min	[100]
		Parathion	LOD: 12.8 nM Linear range: 17 nm–34.4 μM		
	Fluorescence	Paraoxon	LOD: 0.033 fM Linear range: 0.1 fM–0.1 nM	1.5 h	[101]
